# The Effect of Lower Limb Combined Neuromuscular Electrical Stimulation on Skeletal Muscle Cross-Sectional Area and Inflammatory Signaling

**DOI:** 10.3390/ijms252011095

**Published:** 2024-10-16

**Authors:** Amal Alharbi, Jia Li, Erika Womack, Matthew Farrow, Ceren Yarar-Fisher

**Affiliations:** 1 Department of Physical Therapy, College of Applied Medical Sciences, Qassim University, Buraydah 51452, Saudi Arabia; 2 Department of Physical Therapy, University of Alabama at Birmingham, Birmingham, AL 35233, USA; 3 Department of Physical Medicine and Rehabilitation, Ohio State University, Columbus, OH 43210, USA; jia.li@osumc.edu (J.L.); matthew.farrow@osumc.edu (M.F.); 4 Department of Biochemistry, Molecular Biology, Entomology, and Plant Pathology, Mississippi State University, Starkville, MS 39762, USA; ewomack@mscl.msstate.edu; 5 Department of Neuroscience, Ohio State University, Columbus, OH 43210, USA

**Keywords:** spinal cord injury, neuromuscular electrical stimulation, inflammatory signaling, muscle cross-sectional area, myofiber adaptation

## Abstract

In individuals with a spinal cord injury (SCI), rapid skeletal muscle atrophy and metabolic dysfunction pose profound rehabilitation challenges, often resulting in substantial loss of muscle mass and function. This study evaluates the effect of combined neuromuscular electrical stimulation (Comb-NMES) on skeletal muscle cross-sectional area (CSA) and inflammatory signaling within the acute phase of SCI. We applied a novel Comb-NMES regimen, integrating both high-frequency resistance and low-frequency aerobic protocols on the vastus lateralis muscle, to participants early post-SCI. Muscle biopsies were analyzed for CSA and inflammatory markers pre- and post-intervention. The results suggest a potential preservation of muscle CSA in the Comb-NMES group compared to a control group. Inflammatory signaling proteins such as TLR4 and Atrogin-1 were downregulated, whereas markers associated with muscle repair and growth were modulated beneficially in the Comb-NMES group. The study’s findings suggest that early application of Comb-NMES post-SCI may attenuate inflammatory pathways linked to muscle atrophy and promote muscle repair. However, the small sample size and variability in injury characteristics emphasize the need for further research to corroborate these results across a more diverse and extensive SCI population.

## 1. Introduction

Traumatic spinal cord injury (SCI) leads to the disruption of upper motor neuron axons and ascending sensory fibers, culminating in paralysis [1,2]. Skeletal muscle will undergo extensive atrophy, and lower limb muscles are 25–45% smaller than non-injured controls as soon as 6 weeks after SCI, and an additional 20% is lost 24 weeks post-injury [3]. After several weeks to months following SCI, fatigue-resistant and oxidative muscle fibers undergo transformation into highly fatigable and glycolytic muscle fibers [4,5]. However, skeletal muscle that has been paralyzed retains a significant degree of plasticity after SCI [6,7,8,9,10]. The general consensus among available treatments to counteract negative muscle adaptations post-injury suggests that muscle contraction through neuromuscular electrical stimulation (NMES) stands out as the most effective approach. NMES has been widely used to increase paralyzed skeletal muscle mass and function by substituting voluntary contractions [11]. Increasing muscle cross-sectional area (CSA), a measure of muscle size, indicates muscle hypertrophy and overall muscle health [9]. The effects of NMES on skeletal muscle are investigated almost exclusively in people with chronic SCI because of difficulties intervening in the treatment of patients at the inpatient rehabilitation stage [10,11,12,13,14,15].

Therefore, there is a need for novel combined NMES (Comb-NMES) programs that induce key molecular adaptations to both resistance and aerobic exercise to maintain an oxidative, fatigue-resistant muscle phenotype while maintaining or increasing muscle CSA early after SCI. We examined the impact of a novel Comb-NMES program on muscle fiber type distribution and muscle glucose uptake signaling and showed that maintaining healthy myofiber type (i.e., a higher proportion of type I compared to type IIx) and metabolic function may be achieved with the early application of Comb-NMES [16]. Our proposed NMES training program combined dynamic contractions via high-frequency (50 Hz trains of 450 µs biphasic pulses) electrical stimulation (resistance training) with twitch contractions via low-frequency (5 Hz, pulse duration/interval = 200/50 µs) electrical stimulation (aerobic training) on the quadriceps muscle group. Our NMES program induces essential adaptations similar to those achieved through resistance and aerobic exercise, supporting a muscle phenotype that is resistant to fatigue, sensitive to insulin, and optimized for oxidative metabolism [16]. This protocol was feasible for participants to perform comfortably and independently in their wheelchairs or on their hospital beds. Comb-NMES can cause muscle injury similar to those typically associated with voluntary exercises. Although injury may impede rehabilitation and training outcomes, it is now believed that muscle inflammatory responses, if tightly regulated, are vital for muscle repair and regeneration [17]. Understanding the impact of Comb-NMES on inflammatory molecular pathways is essential in developing therapeutic strategies to mitigate excessive inflammation, accelerate muscle repair and regeneration, and slow the progression of muscle atrophy in the acute stage (7–14 days post-injury) of SCI. However, no study has investigated the effect of Comb-NMES on muscle inflammatory signaling and CSA during the acute stages of SCI. Therefore, our purpose in the current project was to determine the effect of Comb-NMES on skeletal muscle CSA and inflammatory signaling within the acute phase of SCI.

The muscle damage following muscle contractions includes disrupted contractile structures and cytoskeletal components [18,19], loss of desmin [20], and permeabilization of the muscle cell plasma membrane [21,22]. The severity of the muscle damage is modulated by the type, intensity, and duration of training. When muscles sustain damage, the process of muscle inflammation initiates through the coordinated activation of various signaling pathways and the mobilization of pro- and anti-inflammatory factors such as macrophages and neutrophils to the injured area. Both recruited and resident immune cells within the damaged muscle release proinflammatory cytokines like Interleukin (IL)-1β and tumor necrosis factor alpha (TNF-α). This initiates a series of subsequent inflammatory signaling pathways, leading to the transformation of early macrophages into an M1 phenotype and the initiation of the ubiquitin–proteasome pathway for protein breakdown. This is achieved by upregulating the gene expression of E3 ligases, such as muscle ring finger 1 (MuRF1) and muscle atrophy F-box (MAFbx or Atrogin-1) [23,24]. The majority of TNF-α’s effects are mediated by upregulating transcriptional activity under the control of nuclear kappa-light-chain-enhancer of activated B cells (NF-κB). NF-κB represents one of the most significant signaling molecules canonically activated by stimulation of proinflammatory factors such as TNF-α or its associated cytokines [25] and the toll-like receptor family (TLR) [26]. Another pathway of interest is the Janus kinase 1 (Jak1)/signal transducer and activator of transcription 3 (STAT3) pathway. In skeletal muscle, activation of the JAK1/STAT3 pathway by the Interleukin 6 (IL-6) cytokines has a dichotomic role: it promotes muscle hypertrophy, by increasing the proliferation of satellite cells, but also contributes to muscle atrophy [27,28,29]. We hypothesized that administering Comb-NMES early after SCI (during the inpatient rehabilitation stage) could prevent or reduce muscle atrophy in the Comb-NMES group compared to a control group by upregulating proteins and/or signaling pathways that are involved in muscle repair and hypertrophy, such as JAK1, STAT3, IL-6, MyoD, S6 ribosomal protein S6, and p70 S6 kinase, and downregulating proteins and/or signaling pathways that are involved in muscle inflammation and atrophy, such as IL-1β, TNF-α, NF-κB, Atrogin-1, TLR, and tumor necrosis factor receptor (TNFR)-associated factor 6 (TRAF6). In addition, we hypothesized that muscle fiber CSA could be maintained or improved in the Comb-NMES group compared to the control group.

## 2. Results

### 2.1. Participants

Nineteen subjects (31 ± 9 years) were included (3 females and 16 males) (Table 1). There were no significant differences between the groups in how demographic variables (injury level, completeness, injury type, gender, and race) were distributed. The control group’s intervention lasted an average of 18 days, while the Comb-NMES group’s lasted 21 days (*p* = 0.38). Comb-NMES subjects were 9.8 years older on average than control group participants (*p* = 0.01). The average amount of electrical current used in the Comb-NMES group for the Dudley and twitch protocols was 123.8 mA, ± 45.2, and 118.2 mA, ± 44.2. The average number of repetitions in the Dudley protocol was 9 ± 2.4.

### 2.2. Skeletal Muscle Intracellular Signaling Proteins

**Figure 1:**
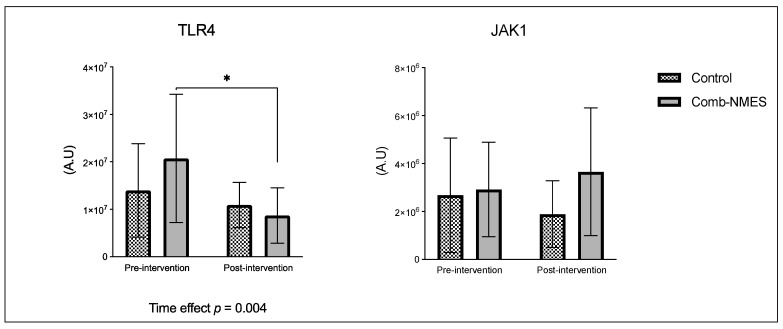

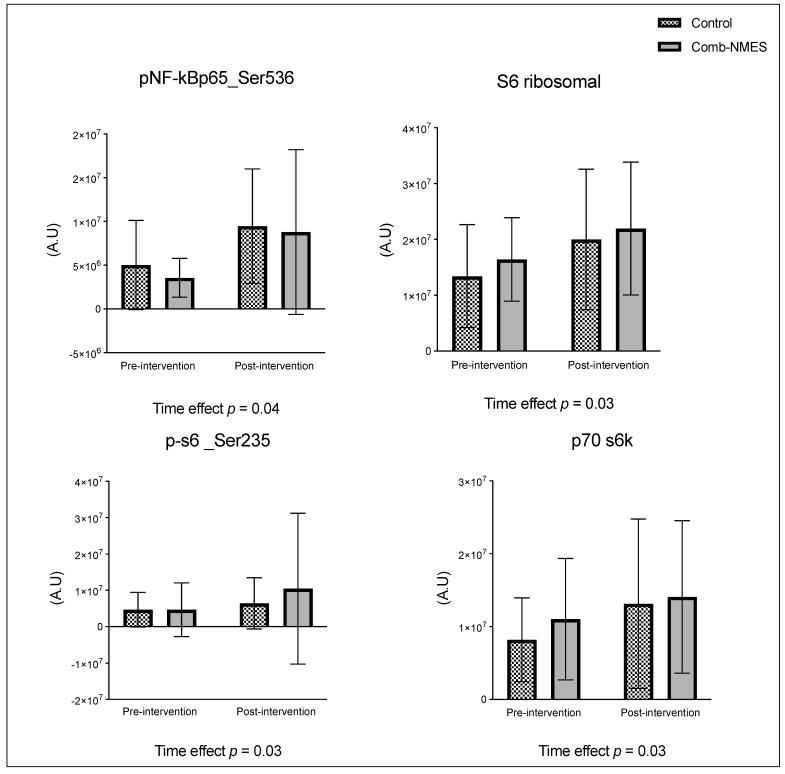

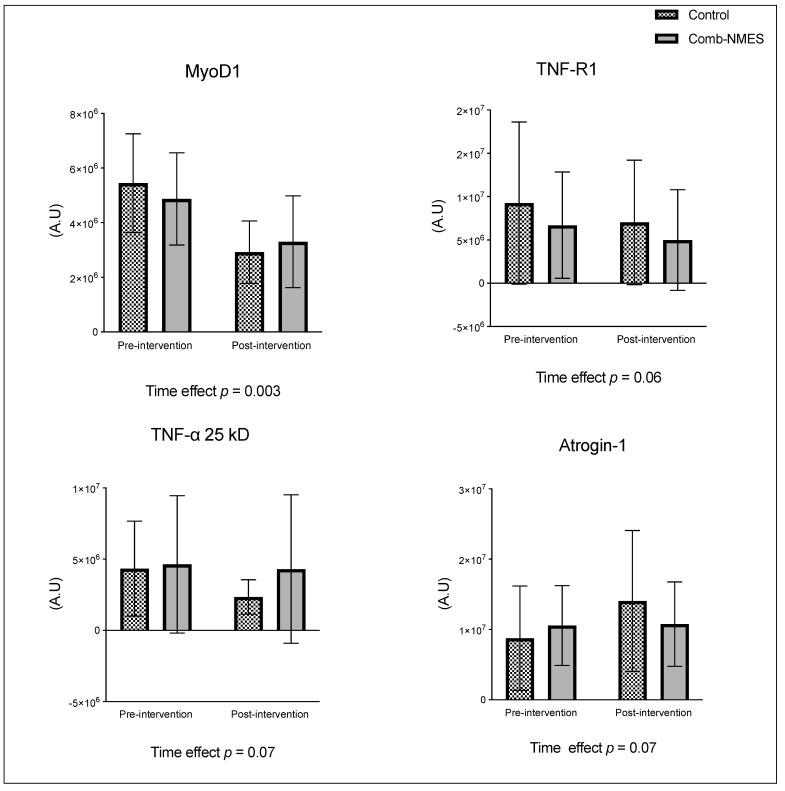

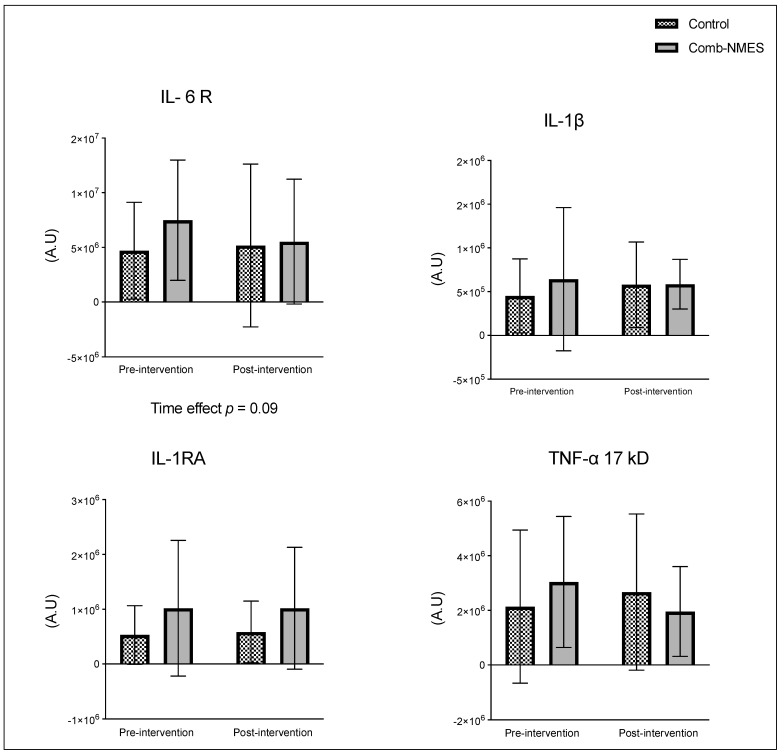

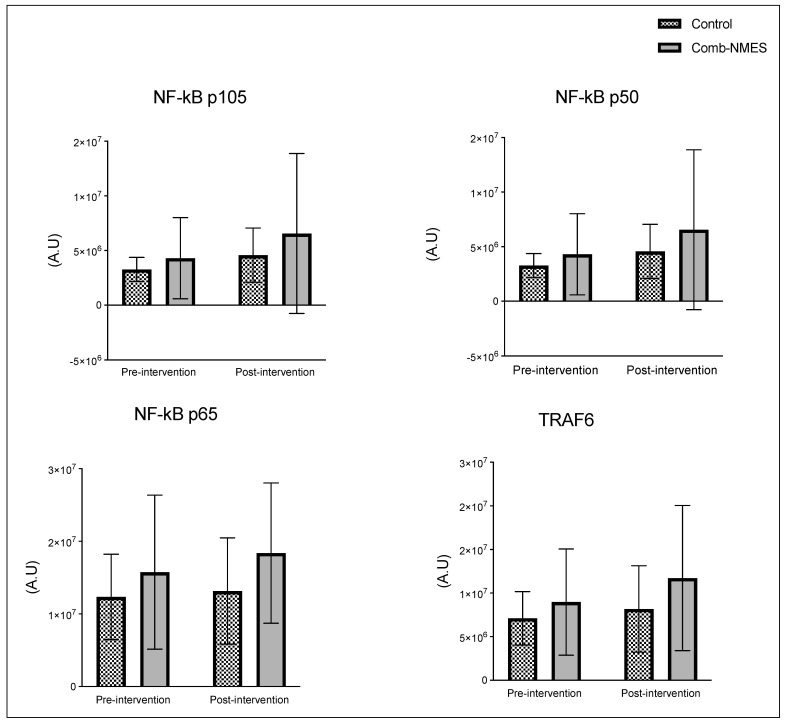

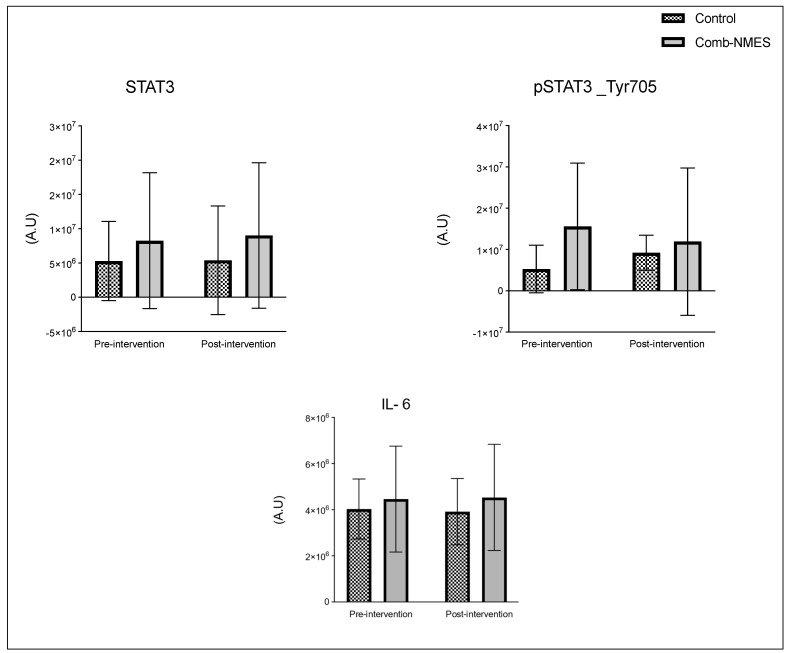
Normalized total protein levels for skeletal muscle inflammatory proteins in response to Comb-NMES vs. control. * Statistically significant changes within the group (*p* < 0.05). Data are presented as means ± SD. Statistical significance was determined using a linear mixed model to assess group–time interactions, followed by pairwise post hoc comparisons using the Tukey–Kramer method. The model assumptions, including homogeneity of variance and normal distribution of residuals, were verified through diagnostic plots. Significance was set at *p* < 0.05; toll-like receptor 4, TLR4; Janus kinase 1, JAK1; nuclear factor kappa-light-chain-enhancer of activated B cells, NF-kB; ribosomal protein S6 kinase, RS6K; myogenic differentiation 1, MyoD1; tumor necrosis factor alpha receptor 1, TNF-R1; tumor necrosis factor alpha, TNF-α; Interleukin 6 receptor, IL-6R; Interleukin 1 beta, IL-1β; Interleukin 1 receptor antagonist, IL-1RA; tumor necrosis factor receptor (TNFR)-associated factor 6, TRAF6; signal transducer and activator of transcription 3, STAT3; Interleukin 6, IL-6.

There were no significant group × time interactions in the proteins assessed (Figure 1). TLR4 and JAK1 exhibited a tendency towards an interaction (group × time) effect (*p* = 0.07, time effect *p* = 0.004, and *p* = 0.09, time effect *p* = 0.9, respectively). TLR4 post hoc analysis revealed that the changes in Comb-NMES were significant (*p* = 0.002, 58% reduction) but not in the control group (*p* = 0.8, 22% reduction). Changes in JAK1 within each group, on the other hand, were not significant (*p* > 0.05). JAK1 increased by 25% in the Comb-NMES group and decreased by 30% in the control group. pNF-kBp65_Ser536, S6 ribosomal, pS6_Ser235, and p70_s6k all increased with time, regardless of treatment group (*p* < 0.05 for time effect), and there were no changes within groups (*p* > 0.05). In addition, MyoD decreased over time regardless of the treatment group (*p* < 0.05 for time effect). Despite the lack of an interaction effect, several proteins increased more in the Comb-NMES group than in the control group. For example, in Comb-NMES, pNF-kBp65_Ser536 and pS6_Ser235 increased by 147% and 123%, respectively, compared to 88% and 37% in the control group. There was a tendency towards a time effect for TNF-R1 (time: *p* = 0.06), TNF-α 25 kD (time: *p* = 0.07), Atrogin-1 (time: *p* = 0.07), and IL-6R (time: *p* = 0.09). Although there was no interaction effect, Atrogin-1 increased by 60% in the control group, while Comb-NMES increased by 2%. Lastly, there was no statistical significance for IL-1β, IL-1RA, TNF-α 17 kD, NF-kB1_p105, NF-kB1_p50, NF-kB_p65, TRAF6, STAT3, pSTAT3_Tyr705, and IL-6 (*p* > 0.05).

### 2.3. Myofiber Size

**Figure 2:**
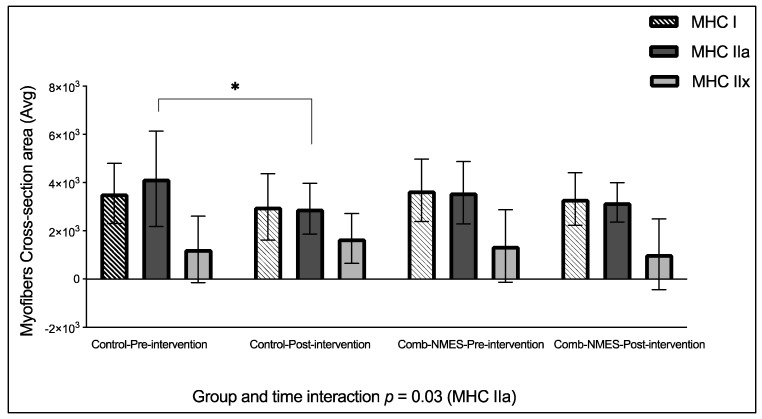
Myofiber cross-sectional area (CSA) in response to the Comb-NMES and control groups. Data are means ± SD. Group × time interactions were evaluated using a linear mixed-effects model. Post hoc Tukey–Kramer tests were performed to determine specific differences between and within groups. Residuals were checked to ensure normal distribution and variance homogeneity. Statistical significance was defined as *p* < 0.05. * Statistically significant changes within the group (*p* < 0.05).

There was a statistically significant group–time effect for MHC IIa CSA, (*p* = 0.03, Figure 2). Post hoc analysis found that the changes in the control group were significant (−30%, *p* = 0.02) but not in the Comb-NMES (−11%, *p* = 0.9). There were no effects of the intervention, time, or their interaction on the CSA of MHC I and MHC IIx fibers (*p* > 0.05). Furthermore, MHC IIx CSA decreased by 25% in the Comb-NMES but increased by 36% in the control group.

## 3. Discussion

Exercise induced by NMES can induce muscle damage, repair, and growth through a complex interplay of molecular and cellular processes [30]. The mechanical stress and strain placed on muscle fibers during muscle contraction can cause microtears in the myofibrils, leading to structural damage and the initiation of the muscle repair process via activation of inflammatory signaling pathways [31]. In addition, the mechanical forces of NMES-induced muscle contraction could initiate the translation required for muscle hypertrophy and growth. This study aimed to investigate the effect of Comb-NMES in the early stages of SCI (14 days after injury) on muscle pathways that contribute to muscle regeneration, repair, growth, and atrophy early after SCI. In addition, we determined the changes in muscle CSA in response to Comb-NMES and sham control treatment (TENS + passive leg exercises).

The main findings of this study included changes ((decrease (−) or increase (+)) in the total level of the following proteins in the Comb-NMES group vs. the control group: TLR4 (−58% vs. −22%), JAK1 (+25% vs. −30%), pSTAT3 (−23% vs. +12%), p-S6_Ser235 (+123% vs. +37%), IL-1β (−9% vs. +28%), TNF-α 17 kD (−35% vs. +24%), and Atrogin-1 (+2% vs. +60%). Both groups exhibit a reduction in the CSA of MHC I and MHC IIa myofibers. However, the reduction in MHC IIa was statistically significant (*p* = 0.03; post hoc analysis: −30%, *p* = 0.02) in the control group but not in the Comb-NMES group (−11%, *p* = 0.9). While the CSA of MHC IIx was reduced in the Comb-NMES group (−25%), it increased in the control group (+36%). The observed reduction in levels of inflammatory/atrophy signaling proteins such as TLR4, pNF-kB p65, TNF-α 17 kD, IL-1β, and Atrogin-1 in the Comb-NMES group, coupled with the increase in muscle repair/growth signaling proteins such as p-S6_Ser235 and JAK1 and smaller reductions in MHC I and IIa muscle fiber CSA in the Comb-NMES group, suggest a multifaceted mechanism by which Comb-NMES may attenuate atrophy soon after SCI.

### 3.1. Skeletal Muscle Intracellular Proteins

Among the known signaling pathways that are involved in skeletal muscle repair and regrowth is the activation of the JAK1/STAT3 pathway [17]. Following muscle contraction, IL-6 is released into the damaged site, which activates the JAK1/STAT3 signaling pathway [17,32]. The activation of the JAK/STAT/IL-6 signaling pathway induces pro-proliferation and pro-fusion genes that control the contribution of muscle satellite cells to myofiber growth, promoting muscle regeneration and growth [33]. The pathway also plays a role in muscle wasting and has a dichotomic effect on myogenic cell proliferation and differentiation [17]. The JAK1/STAT3 pathway is turned on by IL-6 binding to its receptor, IL-6R. IL-6R then forms a complex with the transmembrane protein, glycoprotein 130 (gp130). This complex transmits the IL-6 signal intracellularly, leading to the activation of JAK1. Once activated, JAK1 phosphorylates the cytoplasmic domain of gp130, leading to the recruitment and phosphorylation of STAT3. After undergoing phosphorylation, STAT3 forms dimers and moves to the nucleus. There, it acts as a transcription factor, controlling the expression of specific genes related to diverse cellular processes, such as myogenesis. The JAK1/STAT3/IL6 signaling pathway also helps keep myoblasts from differentiating too soon by stopping the expression of genes like MyoD that are needed for myoblast differentiation and fusion [34]. We observed a 25% increase in JAK1 total protein level in the Comb-NMES group, compared to a 29% reduction in the control group (Figure 1). While STAT3 and IL-6 total protein levels increased in both groups, IL-6R and pSTAT3_Tyr705 increased only in the control group. A decrease in IL-6R in the Comb-NMES group could suggest downregulation of the receptor on the cell surface [34]. Still, the reduction in pSTAT3_Tyr705 is unexpected, as this phosphorylation event is typically associated with STAT3 activation and translocation to the nucleus [35]. We imply that the reduction seen in these markers might represent a temporal sequence in which there is an initial surge in total protein levels, followed by negative feedback mechanisms that downregulate specific components to prevent prolonged activation of this pathway, leading to muscle atrophy. Furthermore, we observed a downregulation of the MyoD total protein level in both groups, potentially due to the influence of the Jak1/STAT3/IL-6 pathway.

The TLR4 signaling pathway plays a vital role in the activation of the transcription factor NF-kB through adapter molecules [32]. The activation of NF-kB by TLR4 is essential for the regulation of inflammatory responses, including the expression of proinflammatory cytokines and the coordination of macrophage transitions during tissue repair and resolution of inflammation [32,36]. However, overexpression of TLR4 inhibits muscle satellite cell proliferation [37]. In addition, when TLR4 is downregulated, it has been reported to contribute to the anti-inflammatory effect of skeletal muscle stem cells [37,38]. Studies have shown that TLR4 deficiency leads to decreased TNF-α expression and reduced inflammatory cell infiltration, indicating a protective role in mitigating excessive inflammation [39]. We showed that the total protein level of TLR4 (Figure 1) decreased in both groups; however, the reduction was significant in the Comb-NMES group. In addition to TNF-α 25kD and TNF-R1, total protein levels also decreased in the Comb-NMES group, which could be influenced by the reduction seen in TLR4.

The total and phosphorylated protein levels of NF-κB in all of its units (p50, 65, and 105) increased in both groups, whereas the increase in pNFκBp65_Ser536 was higher in the Comb-NMES group (+147%) vs. in the control group (+88%). Studies have shown that the p65 subunit is associated with the promotion of myogenesis, indicating a positive regulatory role in skeletal muscle differentiation [40,41,42]. Additionally, the p65 subunit has been identified as containing relatively abundant DNA binding activities in skeletal muscles, suggesting its involvement in the formation and/or function of skeletal muscles [40]. The reduction in TLR4 could indicate an adaptation of the muscle cells to the Comb-NMES intervention, resulting in the resolution of the initial inflammatory response. This adaptation allows the transition to the subsequent stages of myogenesis without sustained activation of TLR4 [39].

The transcription factor NF-κB is not only activated by TLR4, it is also activated by TNF-α and TRAF6 [37,43]. Following muscle damage induced by exercise, proinflammatory cytokines such as TNF-α, IL-1β, and IL-6 are released. These cytokines can activate the NF-κB pathway by binding to their respective receptors on the muscle cell membrane, leading to the activation of downstream signaling molecules [44]. Additionally, the activation of NF-κB by proinflammatory cytokines has been implicated in the inhibition of myogenesis, contributing to impaired muscle regeneration [45,46]. In our results, TNF-α 25 kD and TNF-R1 were reduced in both groups. While total levels of TNF-α 17 kD and IL-1β were reduced in the Comb-NMES group, they increased in the control group. The reduction in these total proteins could be due to the shift from the inflammatory process inducing atrophy to the promotion of hypertrophy and muscle growth. In support of this idea, we observed a notable increase in p-S6 Ser235 in the Comb-NMES compared to the control group. Exercise stimulates the activation of ribosomal proteins S6 kinase, p70 S6K, and p-S6 Ser235 through the mechanistic target of the rapamycin complex 1 (mTORC1) signaling pathway, which plays a pivotal role in regulating protein synthesis and cell growth [47]. Upon activation, mTORC1 phosphorylates and activates p70 S6K, leading to the phosphorylation of the ribosomal protein S6 at multiple serine residues, including Ser235. The phosphorylation of p-S6 Ser235 is a marker of mTORC1 activity and is associated with the initiation of protein synthesis and cell growth in muscle cells [47,48]. The higher increase in p-S6 Ser235 may suggest a promotion of protein synthesis and muscle growth in response to Comb-NMES.

Atrogin-1, often referred to as MAFbx (muscle atrophy F-box), has an important role in the ubiquitin–proteasome pathway during muscle protein degradation [49]. The molecular assessment of muscle protein catabolism uses it as a read-out measurement [36]. We found that Atrogin-1 total protein levels in the control group showed a remarkable 60% increase from the baseline, while the Comb-NMES group displayed only a 2% rise in Atrogin-1 levels. A lower level of Atrogin-1 that is seen after utilizing Comb-NMES suggests a potential protective effect of Comb-NMES on muscle protein degradation.

### 3.2. Myofiber Cross-Sectional Area

After an SCI, there is a substantial risk of muscle wasting due to insufficient muscle use [50]. The occurrence of rapid and extensive muscle mass depletion has been observed within the initial 6 weeks following SCI, and this process persists for a duration of up to 18 months after the injury before reaching a point of stabilization [3]. Muscle fibers in people with SCI tend to change into faster muscle fibers with less aerobic–oxidative enzyme content. This makes the muscles less resistant to fatigue and less able to build force [51]. Atrophying SCI muscles result in a substantial decrease in muscle CSA, ranging from 45% to 80% on average [3]. Luckily, muscle fibers can adjust their characteristics and alter MHC type, structure, and size in reaction to changes in their internal or external surroundings [52]. Activation of paralyzed muscle through NMES has been shown to stimulate muscle hypertrophy [53,54], enhance energy expenditure, and increase force production [53,55]. We found a decrease in the CSA of MHC I and MHC IIa in both groups. However, the reduction in CSA of MHC IIa was significant in the control group (−30%) compared to the Comb-NMES group (−11%) (Figure 2). Furthermore, the Comb-NMES group experienced a reduction in MHC IIx, while the control group experienced an increase. The reduction in myofiber CSA observed in the Comb-NMES group in this study presents a contrast to prior research that demonstrated an increase in myofiber CSA following NMES-RT [6,10,56,57,58]. Crameri et al. [6] applied the NMES-RT protocol to individuals with chronic complete SCI for 30 min, three days a week, over 10 weeks, and found an increase in myofiber CSA. Similarly, Mahoney et al. [10] engaged chronic SCI participants in NMES-RT twice weekly for 12 weeks, using MRI to assess quadriceps CSA, and reported hypertrophic adaptations. Yarar-Fisher et al. [56] implemented a short intervention consisting of 40 actions per day (equivalent to three to four minutes of actual contraction time), three times a week, for eight weeks, and also observed an increase in CSA in people with chronic SCI. Ryan et al. [57] used electrical stimulation under different loads for 45 min, three days a week, for 10 weeks, while another study by Crameri et al. [58] conducted NMES-RT twice weekly for 16 weeks, using dual-energy X-ray absorptiometry as an outcome measure; both studies reported an increase in myofiber CSA. However, the reduction we observed aligns with findings from the study by Crameri et al. [59], which employed NMES aerobic training for 60 min daily, five days a week, over 16 weeks, and reported a slight decrease in myofiber CSA among participants with acute complete SCI. These discrepancies suggest that variables such as intensity, frequency, and protocol duration are critical to eliciting an increase in myofiber CSA with NMES. Additionally, the muscle groups targeted, participant characteristics, and the diversity of outcome metrics in previous research could contribute to the contrasting findings.

Intriguingly, there was an increase in the CSA of the MHC IIx in the control group, whereas a decrease was observed in the Comb-NMES group. The disparity in the CSA could be due to the specific protocol employed. The application of twitch stimulus, akin to aerobic training, may have influenced muscle fiber transformation from type IIX to type IIa or even type I. This type of fiber transformation is conducive to enhanced endurance and metabolic efficiency. We found previously that administration of Comb-NMES to acute SCI participants increased type I myofibers, accompanied by a reduction in type IIx compared to the control group, which showed a reduction in type I myofibers and an increase in type IIx [16]. Additionally, the NMES-RT in the present study follows the Dudley protocol based on increasing the load each session after the first two sessions, which was difficult to achieve in the first week of the intervention. This led to fluctuations in the load that the Comb-NMES participants received. Moreover, the disparities in spasm severity [16], injury level, and completeness among subjects could have impacted the myofiber CSA specifically within the control group. This stresses the complexity of neuromuscular adaptations post-SCI and highlights the necessity to consider individual variability when evaluating the effectiveness of NMES interventions. We published representative fluorescent immunohistochemistry images of myofiber types in our previous work [16].

Although this study focuses on short-term effects, long-term application of Comb-NMES may offer sustained benefits for muscle preservation and metabolic function. Future research should explore whether extended intervention periods yield further improvements in muscle hypertrophy and functional outcomes. Clinically, early application of Comb-NMES post-SCI could be integrated into rehabilitation programs to mitigate early muscle atrophy and support recovery, but larger studies are needed to validate these applications.

### 3.3. Limitations

This study is limited by the small sample size, which may not fully represent the SCI population. Additionally, variability in intervention duration, injury completeness, and spasticity levels may have influenced the observed outcomes, introducing variability into the results. Furthermore, the short duration of the intervention may have limited the potential for muscle hypertrophy, which may require a longer intervention period to manifest.

## 4. Materials and Methods

### 4.1. Ethical Approval

Participants were recruited to a clinical trial, NCT03204240 (registered at clinicaltrials.gov). The study protocol was approved by the University of Alabama in Birmingham’s (UAB) Institutional Review Board, and all procedures were performed in accordance with institutional guidelines. Before enrollment, informed consent was obtained from all participants, which included a full explanation of the study and an opportunity to ask questions.

### 4.2. Study Design and Inclusion Criteria

In a randomized controlled trial, individuals were recruited from the UAB Spain Rehabilitation Center (SRC) in Birmingham, AL. Screening was performed via admission lists and medical charts. Participants aged 18–60 years with a traumatic SCI (C4-L1) sustained within 14 days of injury were enrolled in the study. Participants, classified as American Spinal Injury Association Impairment Scale (AIS) A–C, were medically stable with no history of chronic disease at the time of consent. Participants were assigned using the block randomization method to either the intervention (Comb-NMES) or the control (transcutaneous electrical nerve stimulation (TENS) + passive leg extensions and flexions) group and were blinded to their assigned group. The study began in January 2018 and continued until August 2022. All sessions were completed within 2–5 weeks, during inpatient rehabilitation.

### 4.3. Exercise Training Protocol

All sessions were performed with an experienced trainer. Participants trained three days per week (M, W, F) for 2 to 5 weeks in either a wheelchair or in a bed where both knees were flexed between 70 and 90°. The protocol was administered using the TheraTouch 4.7 (Rich-Mar, Inola, OK, USA) stimulation device with self-adhesive 7.6 × 13 cm electrodes (Axelgaard ValuTrode, Fallbrook, CA, USA) placed over the distal–lateral and proximal–lateral portion of the quadriceps femoris muscle group.

**Figure 3:**
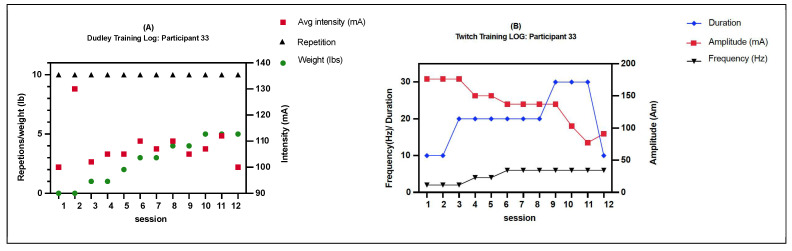
A training program for a person with a complete motor SCI. (**A**) As a component of Dudley’s training (Comb-NMES), every session comprised four sets of ten actions. Initially, during the first two sessions, the individual completed four sets of 10 repetitions without introducing any additional weight. Once the person achieved 40 repetitions of full knee extension within a training session, the weights were gradually increased by 1 lb. Eventually, after completing 12 rounds, the individual was able to lift 5 pounds. (**B**) In the twitch training regimen, the exercise program commenced with 10 min of twitch activation at a frequency of 2 Hz. Over the course of the first week, this duration was gradually extended until each session lasted 30 min, with the frequency increased to 6 Hz [16].

The intervention group received a Comb-NMES exercise, which included NMES resistance exercise (RE) and NMES aerobic exercise (AE). NMES-RE involved concentric and eccentric muscle contractions of the quadriceps. In summary, each session consisted of 4 sets of ten repetitions, stimulated by 50 Hz trains of 450 µs biphasic pulses. To initiate contraction, the current was gradually increased from zero to the desired level (ranging from 50 to 200 mA) within 3 to 5 s. This induced a tetanic muscle contraction, facilitating complete knee extension. Subsequently, the current was decreased over 3 to 5 s to enable the knee to revert to its original flexed position. This cycle was repeated for a total of 5 min and performed on each leg. Ankle weights were progressively increased by 1 lb when the participants were able to complete all sets with full knee extension. For each repeat, the current amplitude of electrical stimulation was checked to make sure that the increase in weight lifted was due to muscle adaptations and not the higher current amplitudes of electrical stimulation (Figure 3A [16]). NMES-AE involved twitch electrical stimulation with a pulse duration of 200 µs and an interval of 50 µs. The current amplitude was set to 175 mA, and the training began with 10 min of twitch stimulation at 2 Hz for the first week on both legs. The duration of the NMES-AE session was progressively increased to 30 min at 6 Hz (Figure 3B [16]). The control group received passive dynamic exercise of the lower legs, mimicking NMES-RE, and TENS, mimicking NMES-AE. An experienced trainer passively moved the participant’s lower legs to complete knee extension and flexion for a similar duration, which was completed with NMES-RE. After completing the passive movement, TENS was set with a pulse duration/intervention of 200/50 between 20 and 60 mA, and its duration was similar to each NMES-AE session. Each group was provided with standard care, which encompassed bed mobility, transfers, wheelchair maneuvering skills, respiratory therapy, management of bowel and bladder functions, control of tone and spasticity, as well as assistance with activities of daily living.

### 4.4. Skeletal Muscle Biopsy Sampling

Following an overnight fast of approximately 10 h, muscle biopsy samples were collected from the vastus lateralis muscle both before and after electrical stimulation training. These biopsy procedures were conducted under local anesthesia (1% lidocaine) utilizing a 5 mm Bergstrom biopsy cannula modified for manual suction. Immediately following the biopsy procedure, muscle fibers of ~50 to 70 mg were cross-sectionally embedded in a donut-shaped mounting medium of tragacanth gum powder/O.C.T. compound (1:2) immobilized on a 1.5 cm^2^ section of cork. The mount was frozen in liquid-nitrogen-cooled isopentane for immunohistochemistry (IHC) analyses. The remaining tissue was snap-frozen in liquid nitrogen in ~30 mg portions for subsequent biochemical assays. All tissue was kept frozen at −80 °C until analysis.

### 4.5. Immunohistochemistry

The frozen muscle mounts underwent cutting into 6 µm cross-sections at −20 °C utilizing a Leica CM1860 Cryostat (Deer Park, IL, USA). These sections were cut in triplicate and then positioned on three-well slides (Electron Microscopy Sciences, Hatfield, PA, USA). Afterward, they were preserved at −80 °C until the immunohistochemistry (IHC) staining procedure. Prior to staining, the cryostat sections were allowed to air-dry at room temperature for 30 min and were maintained in a humidified chamber throughout the staining protocol.

The primary antibodies, NCL-MHCs (anti-MHC I) and NCL-MHCf (anti-MHC II), were sourced from Leica Biosystems (Deerfield, IL, USA), while anti-laminin antibodies were procured from Sigma-Aldrich (St. Louis, MO, USA). Secondary antibodies, ALEXA Fluor 594 (red fluorescent dye) and 488 (bright green fluorescent dye), as well as 3% neutral-buffered formalin, were obtained from Thermo Fisher Scientific (Norcross, GA, USA). Both primary and secondary antibodies were appropriately diluted in 1% goat serum as per the manufacturer’s instructions. Tissue sections were fixed in 3% neutral-buffered formalin for 30 min. Prior to the application of primary and secondary antibodies, sections were blocked with 5% goat serum to minimize non-specific background staining. Subsequently, they were rinsed three times for 5 min each with ice-cold 1× phosphate-buffered saline (PBS; pH = 7.4). Fiber types were distinguished by staining with ALEXA Fluor 594 (for MHC Type I, slow) and ALEXA Fluor 488 (for MHC Type II, fast). For enhanced delineation of fiber boundaries and for determining myofiber size, the anti-laminin antibody was co-stained with ALEXA Fluor 488. Sections were then mounted using Vectashield mounting media with DAPI (Vector Laboratories, Burlingame, CA, USA), resulting in blue fluorescence to highlight nuclei. Subsequently, slides and coverslips were sealed with nail polish and stored at −20 °C until microscopy analysis was conducted.

Staining was visualized using a Nikon Eclipse Ti automated inverted microscope at 10× magnification equipped with NIS-Elements AR v.4 digital imaging software (Nikon, Tokyo, Japan) with florescent filters, DAPI, FITC, and Texas Red. Scanning of the CSA was performed to resolve fine details of the images. For each muscle, the mean fiber CSA was calculated by analyzing ~150 myofibers using NIS Element AR software v.6.1.

### 4.6. Immunoblotting

Frozen skeletal muscle tissue (~30 mg) was pounded into a fine powder using a liquid-nitrogen-chilled Bessman Tissue Pulverizer and homogenized in protein lysis buffer (50 mM Tris-HCl pH 7.4, 150 mM NaCl, 5 mM EDTA, 0.1% SDS, 0.5% deoxycholate, and 1% nonidet P40) supplemented with protease and phosphatase inhibitor cocktails obtained from (Sigma-Aldrich, St. Louis, MO, USA) using the CON-TORQUE homogenizer. Protein lysate was collected from the supernatant after centrifugation at 15,000× *g* for 15 min at 4 °C. Protein concentrations were determined by the Pierce BCA Protein Assay kit (Thermo Scientific, Rockford, IL, USA) according to the manufacturer’s instructions. Total protein was resolved by SDS polyacrylamide gel electrophoresis, and 20 µg samples were transferred onto a 0.45 µm nitrocellulose blotting membrane (Amersham Protran, GE Health Life Science, Chicago, IL, USA) by the Pierce Power Blotter (Thermo Scientific, Rockford, IL, USA) at 25 V, 120 mA, and 17 W for 12 min. The membrane was stained with Ponceau S solution (Sigma-Aldrich, St. Louis, MO, USA) and imaged with the Bio-Rad ChemiDoc Imaging System (version 6.1) on the stain-free gel setting.

Primary antibodies that were purchased from Cell Signaling Technologies included JAK1, MyoD, NF-κB p65, S6 ribosomal protein, phospho-S6 ribosomal protein Ser235, p70 S6 kinase, phospho-NF-κB p65Ser536, NF-κB1 p105/p50, TNF-alpha antibody, STAT3, phospho-STAT3 Tyr705, IL-1β, TNF-R1, TRAF6, and IL-6. TLR4, Atrogin-1, and IL-6R were also acquired for immunoblotting analyses and were purchased from Life Technologies. Primary antibodies were diluted to the manufacturer’s recommended concentrations in 5% bovine serum albumin (see Table 2).

The membranes were treated with non-fat milk (5%) for 1 h at room temperature to block nonspecific binding sites and were then incubated with the primary antibody overnight at 4 °C. Subsequently, the secondary antibody, goat anti-rabbit IgG for horseradish peroxidase (obtained from Fisher Scientific, Rockford, IL, USA, PI32460), was diluted to a concentration of 1:2500 with 5% non-fat dry milk and incubated for 1 h. Protein detection was performed using the SuperSignal West Femto Chemiluminescent Kit (Thermo Scientific, Rockford, IL, USA). Membranes were captured using the Bio-Rad ChemiDoc Imaging System with the chemiluminescent blot setting. The images were analyzed for total protein normalization using Image Lab Software (version 6.1) to ensure accuracy.

### 4.7. Statistical Analysis

Participants’ ages were presented as the mean ± standard deviation (SD). Due to the non-normal distribution of injury duration, we calculated the median and interquartile range (IQR). To compare participant demographics between groups, we utilized unpaired t-tests for age and Mann–Whitney U tests for duration of injury. Fisher’s exact test was applied to compare baseline categorical variables between the groups. The effect of group, time, and their interaction was assessed using a linear mixed-model analysis, with participants treated as a random effect, using the SAS 9.4 proc mixed procedure. Within-group changes were evaluated through pairwise post hoc comparisons using the Tukey–Kramer multiple comparisons method within the linear mixed-effects model. We confirmed statistical model assumptions such as homogeneity of variance and normal distribution of residuals through diagnostic plots. Statistical tests were two-sided, and significance was set at *p* < 0.05. Data were primarily presented as means ± SD unless specified otherwise. Covariates were omitted from the model due to the limited sample size to prevent overfitting of the statistical model. Results from the preliminary analysis including covariables like age, level of injury, completeness, and duration of stay were consistent with the results reported. Muscle samples from four participants were not obtained successfully for analysis, resulting in a final analysis of myofiber size for 15 participants (Comb-NMES: 8; control: 7).

## 5. Conclusions

Our findings suggest that a novel Comb-NMES protocol may influence the size of myofibers and the intracellular signaling related to muscle inflammation and regeneration in individuals with acute SCI. It is important to note that the small sample size and heterogeneity of SCI characteristics limit the generalizability of these findings. While preliminary data suggest potential benefits of early Comb-NMES application, future studies should aim to recruit larger, more diverse populations to enhance the generalizability of their findings. Specifically, increasing the sample size, standardizing the duration and frequency of the intervention, and focusing on more homogenous SCI characteristics (e.g., injury level and completeness) will provide clearer insights into the efficacy of Comb-NMES. Additionally, tracking outcomes over longer intervention periods and including long-term follow-ups will be essential to assess the durability of the observed effects.

It is worth noting that the length of inpatient rehabilitation stays in the US have declined from 98 days in the 1970s to 4–5 weeks at the present time. Similarly, the length of stay varied between 2 and 6 weeks in our participants; therefore, Comb-NMES may induce higher gains in muscle CSA if applied more frequently for a longer duration. Despite the limitations mentioned, the results contribute valuable insights into the multifaceted mechanisms of muscle plasticity post-SCI. They underscore the potential of Comb-NMES to attenuate muscle atrophy and possibly promote muscle repair and regeneration. Our findings lay the groundwork for future research to explore the optimization of NMES protocols and to conduct larger, multi-center trials to substantiate the benefits of Comb-NMES in individuals with SCI. It is hoped that such research will eventually translate into improved clinical practices that enhance muscle health and functional outcomes in this population.

## Figures and Tables

**Table 1:** Characteristics of study participants.

	Age	Sex	Race	AIS	LOI	Complete	Injury Type	Length of Intervention (Days)
Control								
Participant 3	29	Female	Black	A	T2	Complete	GSW	10
Participant 4	20	Male	Black	A	T9	Complete	GSW	11
Participant 16	25	Male	Black	A	L1	Complete	MVC	24
Participant 19	21	Female	Black	A	T9	Complete	GSW	16
Participant 20	20	Male	White	A	T11	Complete	Fall	9
Participant 28	28	Male	Black	A	C6	Complete	GSW	36
Participant 31	26	Male	White	A	T2	Complete	GSW	36
Summary statistics *	25 ± 4 *							18 (10.5–30) *
Comb-NMES								
Participant 1	22	Male	Black	A	C7	Complete	GSW	17
Participant 2	25	Male	White	B	T6	Incomplete	MVC	15
Participant 6	42	Male	White	B	C4	Incomplete	FALL	20
Participant 11	43	Female	White	A	C6	Complete	MVC	23
Participant 12	27	Male	Hispanic	A	T7	Complete	MVC	21
Participant 17	51	Male	Black	C	C3	Incomplete	MVC	39
Participant 22	41	Male	Black	A	C4	Complete	MVC	21
Participant 24	45	Male	White	A	T3	Complete	MCC	28
Participant 29	32	Male	Black	A	T9	Complete	GSW	23
Participant 30	34	Male	White	A	C7	Complete	GSW	21
Participant 33	32	Male	White	B	C4	Incomplete	MVC	24
Participant 34	24	Male	Black	A	T3	Complete	GSW	17
Summary statistics *	35 ± 9 *							21 (18.5–23.5) *

AIS, American Spinal Injury Association Impairment Scale; C, cervical SCI; T, thoracic SCI; LOI, level of injury; GSW, gunshot wound; MVC, motor vehicle collision; MCC, motorcycle crash. * Mean ± SD of age and median (interquartile range) of days in the intervention.

**Table 2:** Antibodies used in immunoblotting and immunohistochemistry.

Antibody	Cat. No.	Dilution	Source
JAK1 antibody	3332S	1:1000	Rabbit
MyoD1 (D8G3)	13812	1:1000	Rabbit
NF-κB p65	8242S	1:1000	Rabbit
S6 ribosomal protein	2217	1:1000	Rabbit
Phospho-S6 ribosomal protein Ser235	4858	1:1000	Rabbit
p70 S6 kinase	34475	1:1000	Rabbit
Phospho-NF-κB p65Ser536	3031S	1:1000	Rabbit
NF-κB1 p105/p50	3035S	1:1000	Rabbit
TNF-alpha antibody	3707S	1:1000	Rabbit
STAT3	4904S	1:1000	Rabbit
Phospho-STAT3 Tyr705	9131	1:1000	Rabbit
IL-1β	12703S	1:1000	Rabbit
TNF-R1	3736S	1:1000	Rabbit
TRAF6	8028S	1:1000	Rabbit
IL-6	12153S	1:1000	Rabbit
TLR4	X1812P	1:500	Rabbit
Atrogin-1	PA5-43915	1:500	Rabbit
IL-6R	PA5-100836	1:1000	Rabbit
Phospho-p70 S6 kinase (Thr421/Ser424) antibody	9204S	1:1000	Rabbit

## Data Availability

The data that support the findings of this study are available on request from the corresponding author [CYF].
